# Clinical Evidence of Chinese Massage Therapy (*Tui Na*) for Cervical Radiculopathy: A Systematic Review and Meta-Analysis

**DOI:** 10.1155/2017/9519285

**Published:** 2017-02-20

**Authors:** Xu Wei, Shangquan Wang, Linghui Li, Liguo Zhu

**Affiliations:** ^1^Department of Scientific Research, Wangjing Hospital, China Academy of Chinese Medical Sciences, Huajiadi Street, Chaoyang District, Beijing 100102, China; ^2^Department of General Orthopedics, Wangjing Hospital, China Academy of Chinese Medical Sciences, Huajiadi Street, Chaoyang District, Beijing 100102, China; ^3^Department of Spine, Wangjing Hospital, China Academy of Chinese Medical Sciences, Huajiadi Street, Chaoyang District, Beijing 100102, China

## Abstract

*Objective.* The review is to assess the current evidence of Chinese massage therapy (*Tui Na*) for cervical radiculopathy.* Methods.* Seven databases were searched. Randomised controlled trials incorporating* Tui Na* alone or* Tui Na* combined with conventional treatment were enrolled. The authors in pairs independently assessed the risk of bias and extracted the data.* Results.* Five studies involving 448 patients were included. The pooled analysis from the 3 trials indicated that* Tui Na* alone showed a significant lowering immediate effects on pain score (SMD = −0.58; 95% CI: −0.96 to −0.21; *Z* = 3.08, *P* = 0.002) with moderate heterogeneity compared to cervical traction. The meta-analysis from 2 trials revealed significant immediate effects of* Tui Na* plus cervical traction in improving pain score (MD = −1.73; 95% CI: −2.01 to −1.44; *Z* = 11.98, *P* < 0.00001) with no heterogeneity compared to cervical traction alone. No adverse effect was reported. There was very low quality or low quality evidence to support the results.* Conclusions. Tui Na* alone or* Tui Na* plus cervical traction may be helpful to cervical radiculopathy patients, but supportive evidence seems generally weak. Future clinical studies with low risk of bias and adequate follow-up design are recommended.

## 1. Introduction

In 1817, Parkinson first observed the clinical feature of cervical radiculopathy [1]. Despite this long history of awareness, cervical radiculopathy caused by degenerative changes remains one of the major contributors for neck pain, commonly seen condition across many patient populations [2, 3]. Pain became a common presenting symptom and might be associated with motor or sensory disorders in areas innervated by the affected nerve root [4, 5]. In addition, neck pain was more prevalent among women and prevalence peaked in middle age [6]. It has been estimated that patients with cervical radiculopathy accounts for 60% to 70% of cervical spondylosis in China [7].

In the absence of myelopathy or obvious muscle weakness all patients with cervical radicular pain should be treated conservatively [8]. At present, the use of conventional modalities for cervical radiculopathy remains debatable, primarily because most treatments had limited success in regard to decreasing levels of pain and disability, increasing function, and range of motion [9–16]. Thus, a general consensus had not been reached for treating cervical radiculopathy worldwide [12]. In response to this situation, a new Chinese clinical consensus of the treatments for cervical radiculopathy was established which will help clinicians to address the common problems in clinical practice [17]. Nevertheless, the Chinese clinical consensus was still limited to a narrow band which mainly provides instructive suggestions to conventional therapeutic methods, including neck immobilization, physiotherapy, analgesic medications, and surgery; further, little is known about the effectiveness of complementary and alternative medicine (CAM) interventions for patients with cervical radiculopathy. In recent years, however, there has been a growing tendency for improving the associated symptoms and clinical signs with CAM therapies [18–20].

One of the sought-after CAM therapies is massage [21, 22]. Massage, dating back to near 2500 BC, is recorded in the oldest existing medical works called* Huangdi Nei jing* in China [23]. There are several types of massage, including but not limited to traditional Chinese massage (known as* Tui Na*), Shiatsu, Thai massage, Swedish massage, reflexology, and myofascial trigger point release [24, 25]. For the patients with neck pain, massage therapy are effective for relieving immediate or short-term pain symptoms, increasing range of motion, and improving neck dysfunction [26–29]. Clinicians and physiotherapists who have been strictly trained usually provide* Tui Na* for the patients with cervical radiculopathy. The operator often uses the finger, hand acting on the muscle, or soft tissue of body parts, based mainly on pain location and muscle tightness. A possible mechanism for the beneficial effects of massage seems to be increased blood flow, relief of muscular spasm, and pain suppression via moderate release *β*-endorphin [30]. As such, massage also can relieve anxiety and depression resulting from cervical radicular neuralgia [31].

Currently, massage is widely applied to treat cervical degenerative disc disease which included cervical radiculopathy [32]. In China,* Tui Na* has been practiced for several years in the treatment of cervical radiculopathy. It involved a wide range of skilled and methodical manipulations best performed by an operator's finger, hand, elbow, knee, or foot applied to muscle or soft tissue at specific parts of the body [33]. Meanwhile, there were a large number of published randomised controlled trials testing the effectiveness of* Tui Na* in patients with cervical radiculopathy. To date, there were no known systematic reviews examining the efficacy of massage, specifically Chinese massage therapy, for the management of cervical radiculopathy. Consequently, this systematic review was undertaken to summarize the clinical evidence of* Tui Na* in the treatment of cervical radiculopathy.

## 2. Methods

The protocol of systematic review was published in the PROSPERO database which was available on https://www.crd.york.ac.uk/PROSPERO/display_record.asp?ID=CRD42016034004 [34].

### 2.1. Types of Studies

All completed randomised controlled trials comparing the efficacy of* Tui Na* for the treatment of cervical radiculopathy were enrolled. The studies reported in abstracts only were also considered, provided that there was sufficient information in the abstract, or available from the author. Animal experiments were not inclusive.

### 2.2. Types of Participants

The clinical diagnosis for cervical radiculopathy was required to be in accordance with the criteria of the North American Spine Society in 2011 (NASS 2011) [2]. If the criterion was not NASS 2011, the diagnosis should be Chinese recognized criteria or definitions from national projected teaching materials.

### 2.3. Types of Interventions

In this review,* Tui Na* was defined as the finger, hand, elbow, knee, or foot applied to muscle or soft tissue surrounding the neck. Randomised controlled trials that evaluated the therapeutic effect of* Tui Na*, including one or more than two types of* Tui Na* methods, compared with no treatment, placebo, or conventional therapies were considered. Combined therapy of* Tui Na* and other conventional interventions compared with other conventional interventions in randomised controlled trials was also enrolled. The interventions containing other CAM treatments (manipulation, mobilization, Chinese herbal medicine, acupuncture, Tai Chi, Wuqinxi exercise, qigong, cupping, etc.) in the* Tui Na* or comparison group were excluded. Multiple publications reporting the same data were also excluded.

### 2.4. Types of Outcome Measures

The primary outcomes were neck and arm pain improvement, intensity of pain evaluated by at least one of the internationally recognized scales such as visual analogue scale (VAS), numerical rating scales (NRS), and McGill pain questionnaire (MGPQ), or similar tools. The secondary outcomes analyzed in this review were neck disability index (NDI), quality of life (SF-36, SF-12) for assessing treatment of cervical radiculopathy recommended by the guideline, or adverse events reported by the included studies [2]. The timing of outcome assessment was defined for four time periods: immediately after treatment (up to one day), short-term follow-up (between one day and three months), intermediate-term follow-up (between three months and one year), and long-term follow-up (one year and beyond) [25, 26, 28].

### 2.5. Information Sources and Search Strategy

We searched seven electronic databases from their inception until January 31, 2016: PubMed, Cochrane CENTRAL, EMBASE, Chinese National Knowledge Infrastructure (CNKI, 1979-), Wanfang database (1998-), Chinese Scientific Journals Database (VIP, 1989-), and Chinese Biomedical Literature Database (CBM, 1978-). These Chinese databases included medical and physical therapy, traditional Chinese medicine (TCM) articles, and full manuscripts plus conference proceedings which could be retrieved. The search terms included “cervical radiculopathy”, “cervical spondylotic radiculopathy”, “nerve-root-type cervical spondylosis”, “cervicobrachial pain”, “cervicobrachialgia”, “neck and arm pain”, “brachialgia”, “brachial neuralgia”, “brachial plexus neuropath^*∗*^”, “neck pain with radiculopathy”, or “neck disorder with radiculopathy” combined with “massage”, “Chinese massage”, “Tui Na”, “TuiNa”, “manual therapy”, and “chiropractic”. There was no limitation on language, publication type, and status. Reference lists of review articles and included trials were searched.

The strategy for searching PubMed was listed as follows:

((((((((((cervical radiculopathy [Title/Abstract]) OR cervical spondylotic radiculopathy [Title/Abstract]) OR nerve-root-type cervical spondylosis [Title/Abstract]) OR cervicobrachial pain [Title/Abstract]) OR cervicobrachialgia [Title/Abstract]) OR (neck [Title/Abstract] AND arm pain [Title/Abstract])) OR brachialgia [Title/Abstract]) OR brachial neuralgia [Title/Abstract]) OR brachial plexus neuropath^*∗*^ [Title/Abstract]) OR neck pain with radiculopathy [Title/Abstract]) OR neck disorder with radiculopathy [Title/Abstract]
(((((massage [Title/Abstract]) OR Chinese massage [Title/Abstract]) OR Tui Na [Title/Abstract]) OR TuiNa [Title/Abstract]) OR manual therapy [Title/Abstract]) OR chiropractic [Title/Abstract]
(1) AND (2)

### 2.6. Study Selection

Two authors independently searched and assessed all the retrieved studies in an unblinded standardized manner. During title and abstract screening, relevant studies were saved. Then the studies with accessible full text were retrieved and further assessed according to the eligibility criteria. The PRISMA flow diagram was used to record the selection processes. Any disagreement was resolved through discussion or, if required, we consulted a third review author.

### 2.7. Data Extraction and Management

Data collection was independently conducted by two authors. For eligible studies, two review authors extracted the data using the agreed form. We extracted the following information: (1) the first author or the first two authors' names and year of publication; (2) basic characteristics of the studies—sample size, diagnosis criteria, intervention characteristics (duration and number of treatment sessions), and outcome measures; (3) basic characteristics of the patients—population characteristics (age, sex), baseline, and before and after treatment; (4) the detailed description of* Tui Na* techniques. When information regarding any of the above was unclear, we contacted authors of the original studies.

### 2.8. Risk of Bias

Two authors assessed the methodological quality using the criteria outlined in the* Cochrane Handbook for Systematic Reviews of Interventions* [40]. And two authors compared the results and discussed difference according to the Cochrane criteria until agreement was reached. The domains included random sequence generation, allocation concealment, blinding of participants and personnel, blinding of outcome assessment, incomplete outcome data, selective reporting, and other bias. For other sources of bias, two aspects were identified: (1) a trial stopped early owing to some data-dependent process; (2) the baseline was extreme imbalance. We reported the judgment for each criterion as “yes (low risk of bias),” “no (high risk of bias),” or “unclear (information is insufficient to evaluate)”.

### 2.9. Data Synthesis

We carried out data analysis using the Review Manager 5.2.0 software. For all outcomes we conducted the analysis based an intention-to-treat principle. For the continuous data, mean difference (MD) was used to assess the difference in the same way between the groups. Standardized mean difference (SMD) was chosen if clinical outcome was the same but measured using different methods in the different trials. *I*^2^ ≥ 50% was identified moderate or significant heterogeneity. And the 95% confidence intervals (CI) were calculated in the meta-analysis. The random-effect model was used to calculate the treatment effect across trials when substantial heterogeneity existed. Where there are high levels of heterogeneity we would advise caution in the interpretation of results.

### 2.10. Quality of Evidence

The overall quality of evidence was assessed for each important outcome using the grading of recommendations assessment, development, and evaluation (GRADE) approach [41–45]. Levels of quality of evidence recommended by the GRADE Working Group were defined as high, moderate, low, and very low [46, 47].

## 3. Results

### 3.1. Search Strategy

**Figure 1:**
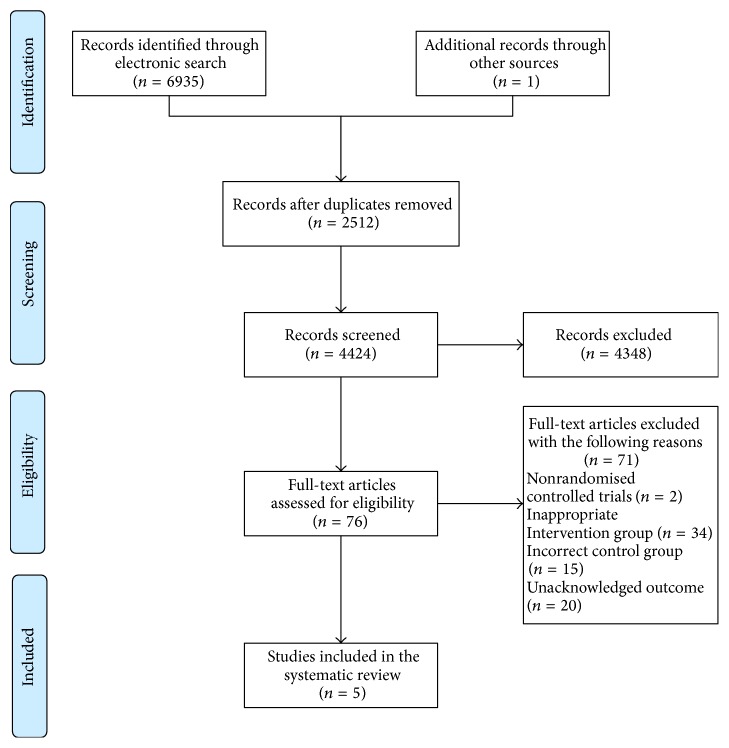
Flow diagram of the literature searching and study selection.

The electronic search found a total of 6935 titles and abstracts. One additional clinical study was identified from a conference proceeding. The full text of 76 articles was retrieved and assessed the studies for inclusion in the review. As a result of limitation in the inclusion criteria we excluded 71 studies: nonrandomised controlled trials (*n* = 2), inappropriate intervention group (*n* = 34) such as* Tui Na* combined with manipulation/mobilization, incorrect control group (*n* = 15) including CAM interventions (Chinese herbal medicine, acupuncture, moxibustion, cupping, plaster for external use, iontophoresis of TCM, and functional exercise), unacknowledged outcomes (*n* = 20) covering self-compile assessment scale, and clinical effect evaluation criteria which were not reported in the international guideline or expert consensus. A total of 5 reports involving 448 patients with relevant outcome data were eligible for inclusion. All included trials were conducted in China and published in the Chinese journals from 2011 to 2014 [35–39]. See Figure 1 for summary of search results.

### 3.2. Patient Characteristics

One study used the diagnosis criterion of State Administration of Traditional Chinese Medicine in 1994 (SATCM-1994) [37]; another used the criterion from National Projected Teaching Materials of China in 2007 (NPTMC-2007) [38], while the other studies applied the criterion issued by Chinese Medical Association in 1993 (CMA-1993) [35, 36, 39]. However, the three criteria used to diagnose cervical radiculopathy were almost the same for the main symptoms (neck and arm pain, paresthesias, numbness, and sensory changes) and signs (Spurling test, Jackson test, and Eaton test) and imaging examination (cervical spine X-ray photograph, computed tomography, or magnetic resonance imaging) compared with the NASS criteria. The three diagnosis criteria were depicted for each study in the characteristics of included studies as shown in Table 1. Participants were generally adult patients over 45 years of age, explicitly excluding patients with myelopathy or obvious muscle weakness. The prevalence of women participants was higher than that of men. No significant difference on baseline was identified in all the studies. The basic characteristics of included participants were described in Table 2. Of the studies which had outcomes included in the meta-analysis (*n* = 228/220), the main intervention strategies were categorized as* Tui Na* (*n* = 138),* Tui Na* plus cervical computer traction (*n* = 90), and cervical computer traction (*n* = 220). Cervical computer traction meant that the subjects received mechanical intermittent cervical traction guided by computer programme. In three studies the intervention was classified as “*Tui Na*” as the arm received only cervical computer traction [35, 37, 39]. In two studies, the primary aim of the study was to compare the effect of* Tui Na* plus cervical computer traction with cervical computer traction [36, 38]. The treatment duration of traction ranged from 15 to 30 minutes once every other day or once a day in the trials. In clinic application, the traction load was mainly determined by different individuals' weight and pain levels. These studies were reported as separate comparisons with cervical traction and only pain score outcomes were included [35–39]. All the treatment duration of the included studies was beyond 2 weeks. In our review, we summarized the massage techniques as shown in Table 3.

### 3.3. Risk of Bias

Table 4 showed the summary of methodological quality, respectively. In the included studies the method used to generate the randomisation sequence was not described or was not clear. Only one study reported allocation by completely random number table, implying random sequence generation [38]. We did not identify the methods of concealing the study group allocation in the studies. All the studies included in the review were not placebo-controlled. In the physiotherapy study, blinding might not have been convincing to patient and clinical staff. The blinding of outcome assessment was not reported in the trials. The amount of incomplete outcome data was generally low, with attrition levels below 5%. But all the trials did not report the follow-up data on the some outcome. Although the studies provided some data on pain assessment, information on other outcomes was sparse, such as quality of life. None of the studies stopped early. Baseline imbalance was not found in the demographic characteristics or the outcomes between the study groups.

### 3.4. *Tui Na* versus Cervical Computer Traction for Pain Score

**Figure 2:**
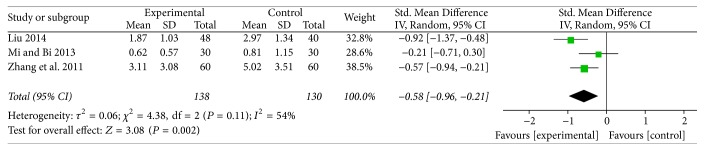
Forest plot of the comparison of* Tui Na* versus cervical computer traction for pain score.

Three studies compared* Tui Na* to cervical computer traction for the outcome of pain score [35, 37, 39]. Results from the pooled analysis indicated that* Tui Na* showed a significant lowering immediate effects on pain score (SMD = −0.58; 95% CI: −0.96 to −0.21; *Z* = 3.08, *P* = 0.002) in cervical radiculopathy patients (Figure 2). A random-effects model was used for statistical analysis according to the moderate heterogeneity (*I*^2^ = 54%). SMD was chosen because pain score was measured by different tools in the different trials; one was NRS [35] and the other two studies used VAS [37, 39]. For the pain measures, however, the difference of change scores between the intervention and control groups should reach the 2 points that was generally accepted as clinically meaningful [24]. So the result of meta-analysis did not support the clinical significance of* Tui Na* therapy.

### 3.5. *Tui Na* Plus Cervical Computer Traction versus Cervical Computer Traction for Pain Score

**Figure 3:**
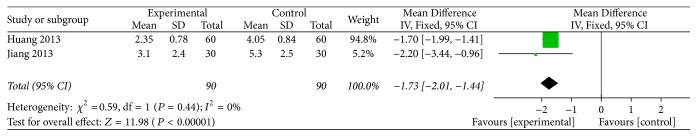
Forest plot of the comparison of* Tui Na* plus cervical computer traction versus cervical computer traction for pain score.

Two studies compared* Tui Na* plus cervical computer traction to cervical computer traction for the outcome of pain score [36, 38]. The meta-analysis from the 2 independent trials revealed significant immediate effects of the combination therapy in improving pain score (MD = −1.73; 95% CI: −2.01 to −1.44; *Z* = 11.98, *P* < 0.00001) (Figure 3) for cervical radiculopathy patients. A fixed-effects model was used to analyze the data with no heterogeneity (*I*^2^ = 0%). Similarly, the result only suggested a trend in favour of* Tui Na* therapy rather than clinical significance.

### 3.6. Adverse Effects of* Tui Na*

Adverse effects were poorly reported in the studies. As shown in Table 1, no trial paid attention to the side effects from treatment or adverse events for the participants.

### 3.7. Publication Bias

For the same intervention and outcome, funnel plot analysis could not be conducted due to the small number of included studies (less than 10) in the meta-analysis.

### 3.8. Quality of Evidence

According to the GRADE approach, each comparison for the same outcome was assessed. When* Tui Na* is compared to cervical computer traction, very low quality evidence (3 trials, 268 participants) was identified to support the effect of* Tui Na* alone for improving the neck and arm pain. We downgraded the quality rating by three levels, including the limitations in study design and execution, inconsistency of results, and imprecision. When* Tui Na* plus cervical computer traction is compared to cervical computer traction, low quality evidence (2 trials, 180 participants) was identified to confirm the effect of* Tui Na* plus cervical computer traction for relieving the neck and arm pain. The quality of evidence for this outcome was low because of the previous studies with high risk of bias and imprecise result.

## 4. Discussion

### 4.1. Summary of Main Results

Massage is one of the frequently applied nonoperative interventions to relieve the symptoms of cervical radiculopathy [48]. However, evidence to confirm the efficacy of massage including* Tui Na* for cervical radiculopathy is scarce. This systematic review included 5 studies that investigated the effect of* Tui Na* alone or* Tui Na* plus cervical computer traction on cervical radiculopathy patients. All the primary studies were from China. The treatment duration was within 1 month in the studies. All the randomised studies reported a reduction in pain scores between the groups.

These results suggested that* Tui Na* or the combination therapy alleviated the pain symptom and that the focus should lie on the immediate effects on pain score. According to the results of meta-analysis, the highest reduction in pain scores was associated with the combination therapy. But the difference between the intervention and control groups did not reach what is generally considered the minimally clinically important difference. This review included no studies to investigate the short-, intermediate-, and long-term effect of* Tui Na*. From the* Tui Na*'s safety point of view, we did not determine the adverse event or adverse drug reaction in the course of treatment so far. However, there was no high quality of evidence to support the effect of* Tui Na*, whether* Tui Na* alone or* Tui Na* plus cervical computer traction in the treatment of cervical radiculopathy on the basis of GRADE approach.

### 4.2. Comparison with the Literature

*Tui Na* used alone or combined with analgesics drugs or cervical computer traction has been widely used as adjunctive treatment for cervical radiculopathy in China. And until now two systematic reviews reported the effectiveness or safety of* Tui Na* or manipulation for cervical radiculopathy [49, 50]. However, a systematic review has not been evaluated based on the PRISMA statement [49]. The other systematic review confirmed the potential benefit and safety of massage or manipulation, but only enrolling cervical computer traction as a control group [50]. Even more important, the interventions which also include high-velocity and low-amplitude manipulation do not belong to massage in the two systematic reviews. Therefore, neither of the systematic reviews has commented on the role of Chinese massage therapy in the treatment of cervical radiculopathy.

### 4.3. Strengths and Limitations

As we all know, this is the first systematic review which analyzes the effect of* Tui Na* for cervical radiculopathy. According to the results of current literatures, we have come to the tentative conclusion about the effect of* Tui Na* alone or* Tui Na* combined with cervical computer traction.

Nevertheless, our study had several limitations. First of all, the included studies used a relative small number of patients, varying between 60 and 120 patients. None of the studies performed a power calculation. Further, multiple diagnosis criteria might introduce some bias into the study. In the second place, there was a lack of well-designed randomised controlled trials. For example, concrete random method and allocation concealment were unclear. The majority of the studies were rated “unclear” or “high” risk of bias with regard to blinding. Generally, results need to be downgraded as to level of effectiveness as no blind placebo-controlled trials were included in the meta-analysis. And no dose-response study design was found in all the literatures. In the third place, our review clearly described massage techniques as stand-alone treatments without cervical manipulation or mobilization techniques, but there is the lack of consistent terminology for massage therapy, as reported by others [51, 52]. Fourthly, the included scales were mainly used to evaluate the variation of pain, whereas other scales in relation to cervical spine function or quality of life were not enrolled. Last but not the end, the previous articles were limited related to adverse events in the studies. However, the known or expected adverse effects of* Tui Na* might include dizziness, nausea, increasing pain in the clinical practice and palpitation, and headache reported in the literature [53].

## 5. Conclusion

In conclusion, there was weak endorsement of* Tui Na* alone or* Tui Na* plus cervical computer traction for cervical radiculopathy patients. And the safety of* Tui Na* could not yet be judged. Based on the available randomised trials, there is a lack of strong evidence for* Tui Na* alone or* Tui Na* plus cervical computer traction according to GRADE approach. As a result, there is a need for well-designed randomised controlled studies with sufficient power so as to confirm the effectiveness of* Tui Na* to improve the clinical manifestation, including the pain symptom, cervical function, and quality of life.

## Figures and Tables

**Table 1:** Basic characteristics of the included trials.

Reference	Sample size	Diagnostic criteria	Intervention	Control	Treatment duration	Outcome measures
Zhang et al. 2011 [35]	120	CMA-1993	*Tui Na* (once every other day)	Cervical computer traction (20 minutes, once every other day)	20 days	Pain score (NRS)
Jiang 2013 [36]	60	CMA-1993	*Tui Na* (once a day) + C	Cervical computer traction (30 minutes, once a day)	2 weeks	Pain score (VAS)
Mi and Bi 2013 [37]	60	SATCM-1994	*Tui Na* (once a day)	Cervical computer traction (20–30 minutes, once a day)	2 weeks	Pain score (VAS)
Huang 2013 [38]	120	NPTMC-2007	*Tui Na* (once a day) + C	Cervical computer traction (15 minutes, once a day)	28 days	Pain score (VAS)
Liu 2014 [39]	88	CMA-1993	*Tui Na* (once every other day)	Cervical computer traction (20 minutes, once a day)	2 weeks	Pain score (VAS)

CMA-1993 = diagnosis criterion issued by Chinese Medical Association in 1993; SATCM-1994 = diagnosis criterion of State Administration of Traditional Chinese Medicine in 1994; NPTMC-2007 = diagnosis criterion from National Projected Teaching Materials of China in 2007; C = control group; NRS = numerical rating scale; VAS = visual analogue scale.

**Table 2:** Basic characteristics of the included subjects.

Reference	T/C (M/F)	Age (yrs)	Baseline difference	Pain scores	
				BT	AT
Zhang et al. 2011 [35]	T/C: 60/60 M/F: 48/72	Total population: 18–35 M/F: NR	NSD	T: 8.67 ± 5.02 C: 8.87 ± 5.27	T: 3.11 ± 3.08 C: 5.02 ± 3.51
Jiang 2013 [36]	T: 30 (18/12) C: 30 (16/14)	T: 51 ± 6.7 C: 48 ± 7.1	NSD	T: 7.2 ± 2.6 C: 7.5 ± 2.3	T: 3.1 ± 2.4 C: 5.3 ± 2.5
Mi and Bi 2013 [37]	T: 30 (14/16) C: 30 (13/17)	T: 47.5 C: 45.6	NSD	T: 4.53 ± 2.16 C: 4.56 ± 1.80	T: 0.62 ± 0.57 C: 0.81 ± 1.15
Huang 2013 [38]	T: 60 (31/29) C: 60 (26/34)	T: 45.62 ± 11.64 C: 45.34 ± 12.06	NSD	T: 6.35 ± 1.11 C: 6.43 ± 1.05	T: 2.35 ± 0.78 C: 4.05 ± 0.84
Liu 2014 [39]	T: 48 (20/28) C: 40 (16/24)	T: 51.23 ± 5.20 C: 51.54 ± 5.15	NSD	T: 5.93 ± 1.08 C: 5.98 ± 1.01	T: 1.87 ± 1.03 C: 2.97 ± 1.34

T = treatment group, C = control group, NR = no reported, M = male, F = female, yrs = years, NSD = no statistical difference, BT = before treatment, and AT = after treatment.

**Table 3:** Treatment with massage techniques in intervention group.

Reference	Treatment with massage techniques
Zhang et al. 2011 [35]	(1) Press and knead the soft tissue around the neck and shoulder using the thumb (the patient is in prostrate position) (2) knead the acupoints: Fengchi (GB20), Fengfu (GV16), and Ashi points using a moderate force (the patient is in supine position) (3) Stretch and rotate the neck (the patient is in supine position) (4) Press and knead the neck and shoulder, followed by grasping the shoulder (the patient is in sitting position)

Jiang 2013 [36]	(1) knead and palm-rub the neck and shoulder muscle (the patient is in sitting position all the time) (2) Press and knead the acupoints: Fengchi (GB20), Tianzhu (BL10), Tianzong (SI11), Dazhui (GV14), Jianjing (GB21), Fengmen (BL12), Houxi (SI3), and Hegu (LI4) (3) Stretch and rotate the neck (4) knead the neck and shoulder muscle again

Mi and Bi 2013 [37]	(1) knead and roll cervical spinous process and paraspinous tissue (2) Grasp and knead the acupoints and meridians in the patient's affected upper extremities: Jianjing (GB21), pericardium meridian, triple energizer meridian, large intestine meridian, and small intestine meridian (3) Roll the acupoints: Tianzong (SI11) and Zhongfu (LU1) using metacarpophalangeal joints (4) Press the acupoints: Futu (LI18), Tianding (LI17), Quchi (LI11), Hegu (LI4), Yangxi (LI5), and Yanggu (SI5)

Huang 2013 [38]	(1) Grasp and knead the soft tissue around the neck and shoulder and press and knead the acupoints: Tianzong (SI11), Jianjing (GB21), and Dazhui (GV14); pull and stretch the upper extremities (the patient is in supine position all the time) (2) Hold the distal end of the patient's affected upper extremities and shake in small amplitude (3) Rap the soft tissue around the neck and shoulder (4) Pat the back using the hollow palm

Liu 2014 [39]	(1) Grasp and knead the acupoints: Fengfu (GV16), Fengchi (GB20), Tianding (LI17), Tianzhu (BL10), Jianjing (GB21), Quepen (ST12), Quchi (LI11), Xiaohai (SI8), Shousanli (LI10), Waiguan (TE5), Neiguan (PC6), Shenmen (HT7), and Hegu (LI4) (the patient is in sitting position all the time) (2) Roll, grasp, and knead the muscle around the neck and shoulder; especially press and knead the scalene muscle and pressure point (3) Stretch and rotate the neck (4) Grasp and knead the acupoints: Jianjing (GB21); hold the distal end of the patient's affected upper extremities and shake in small amplitude and pat the back and the upper extremities

**Table 4:** Methodological quality of the included trials based on the Cochrane Handbook.

Reference	A	B	C	D	E	F	G
Zhang et al. 2011 [35]	?	?	−	?	+	?	+
Jiang 2013 [36]	?	?	−	?	+	?	+
Mi and Bi 2013 [37]	?	?	−	?	+	?	+
Huang 2013 [38]	+	?	−	?	+	?	+
Liu 2014 [39]	?	?	−	?	+	?	+

A = random sequence generation; B = allocation concealment; C = blinding of participants and personnel; D = blinding of outcome assessment; E = incomplete outcome data; F = selective reporting; G = other bias; “+”, low risk of bias; “−”, high risk of bias; “?”, unclear risk of bias.

## Abbreviations

CAM: Complementary and alternative medicine
NASS: North American Spine Society
VAS: Visual analogue scale
NRS: Numerical rating scales
MGPQ: McGill pain questionnaire
NDI: Neck disability index
CNKI: Chinese National Knowledge Infrastructure
VIP: Chinese Scientific Journals Database
CBM: Chinese Biomedical Literature Database
MD: Mean difference
SMD: Standardized mean difference
CI: Confidence intervals
GRADE: Grading of recommendations assessment, development, and evaluation
SATCM: State Administration of Traditional Chinese Medicine
NPTMC: National Projected Teaching Materials of China
CMA: Chinese Medical Association.
